# Photopheresis Provides Significant Long-Lasting Benefit in Nephrogenic Systemic Fibrosis

**DOI:** 10.1155/2017/3240287

**Published:** 2017-06-12

**Authors:** Ranran Zhang, William Nicholas Rose

**Affiliations:** Department of Pathology and Laboratory Medicine, University of Wisconsin, Madison, WI, USA

## Abstract

Nephrogenic systemic fibrosis (NSF), previously known as nephrogenic fibrosing dermopathy, is a rare complication of exposure to gadolinium-based contrast agents in patients who have significantly decreased renal function. Manifestations include fibrosis of the skin and other tissues. Effective therapies are lacking. Photopheresis has been tried with variable rates of improvement, and small numbers of cases (20 as of 2016) have been reported of NSF patients treated with photopheresis. We report a case of patient with nephrogenic systemic fibrosis who was treated with photopheresis and demonstrated significant lasting improvements.

## 1. Introduction

Nephrogenic systemic fibrosis is a rare but well-recognized severe systemic complication of gadolinium-based contrast agents. It occurs exclusively in patients with renal insufficiency [1, 2]. The pathophysiology is emerging. A process similar to wound healing driven by proinflammatory and profibrotic pathways may be one of the underlying causes [1]. Extracorporeal photopheresis (ECP) is a treatment method that improves several autoimmune or inflammatory conditions. Scattered case reports and case series suggest that ECP improves symptoms in patients with NSF [2].

The 2016 evidence-based guidelines from the American Society for Apheresis state that outcomes have been reported for 20 patients who were treated with ECP for NSF [3]. Thus, due to the very small number of reports, our goal is to contribute another data point, however meager, in an effort to help practitioners manage these patients.

Furthermore, coverage decisions typically depend on published evidence or the lack thereof. Due to the expense of photopheresis, the treatment is often highly scrutinized by Medicare in the United States, and coverage denials are very common. We advocate for a reconsideration of summary coverage denials of ECP for NSF.

## 2. Case Report

A 60-year-old male presented with progressive fibrotic indurated skin plaques, multiple contractures, severely limited range of motion of all limbs, and joint pain. Symptoms started while he was recovering from an episode of severe sepsis four years prior to presentation.

His sepsis at that time (four years prior to presentation) was complicated by acute renal injury requiring temporary hemodialysis and cervical spine epidural abscess causing paraplegia.

At that time (four years prior to presentation), multiple MRI studies with gadolinium-based contrast agents were performed, while he was on hemodialysis. The combination of gadolinium exposures in concert with dialysis dependence was most likely the cause of his NSF.

**Figure 1:**
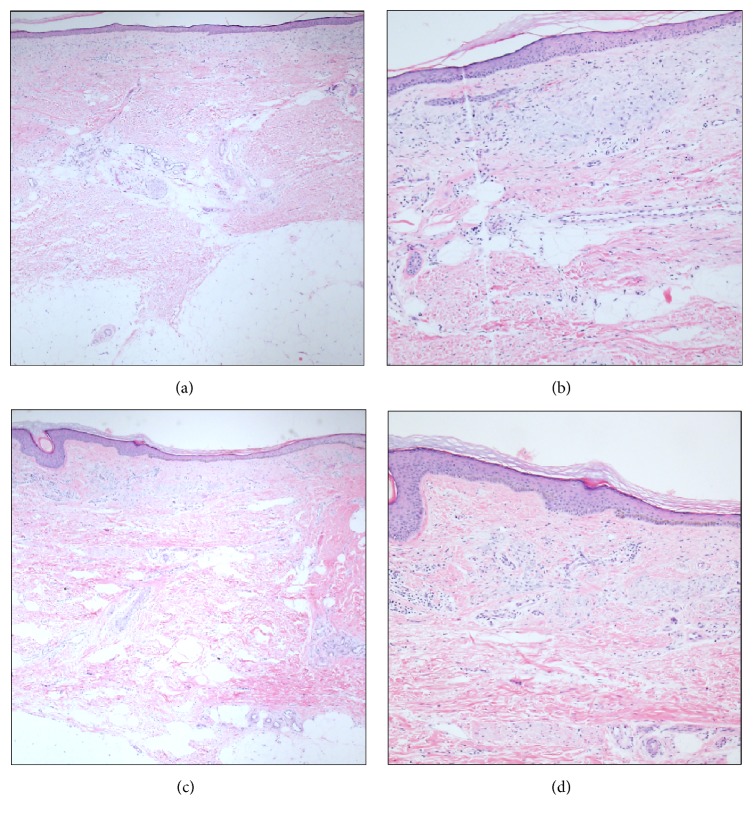
Skin punch biopsies before ((a) at 4x magnification; (b) at 10x magnification) and after ECP ((c) at 4x magnification; (d) at 10x magnification). Despite clinical improvement, interval changes in histology were not dramatic. Fibrocollagenous thickening of dermis was seen in both biopsies. In addition, dermal perivascular plasmacytic infiltrates were minimal in both biopsies.

At the time of presentation, the diagnosis of NSF was based on clinical suspicion (i.e., the aforementioned plaques, indurations, and joint contractures in the setting of gadolinium exposure while being dialysis-dependent). Skin punch biopsy performed at presentation was noteworthy for increased cellularity, thick and thin collagen fibers, and elastic preservation (Figures 1(a) and 1(b)).

After a one-month trial of sodium thiosulfate without improvement, he was started on ECP using the Therakos UVAR XTS for two consecutive days (i.e., one cycle) per week for four weeks (i.e., eight total ECP procedures over four weeks). The patient reported and showed improvement as early as one week after the completion of this first batch of four cycles of ECP. Bilateral knee joints contractures and reduced range of motion (ROM) were the main limitations of patient's mobility and their improvement was relatively well-documented.

This initial 4-week course was based on ASFA's guidelines for using ECP in a variety of diseases such as cutaneous T-cell lymphoma, hematopoietic stem cell transplant associated graft-versus-host disease, and cellular rejection of lung transplant [3]. This starting protocol was also what others reported in case reports that described ECP as a treatment for NSF [4, 5].

A very common principle of using ECP is to start with this schedule initially and then taper to every 2 weeks times 4-5 cycles and then every month times 3 cycles (and continue as needed depending on response).

We must emphasize that the precise quantity of cycles beyond the initial 4-week period was relatively arbitrary and guided by clinical judgment and prudence for patient finances since there was no ASFA guideline for ECP in NSF at the time and because there was uncertainty about reimbursement coverage.

After the initial four cycles, a three-month interval passed without ECP. An important reason for pausing ECP at that time was concern about reimbursement coverage. ECP was then restarted with a schedule of one cycle every two weeks for six cycles. This was then tapered to one cycle every month for seven cycles. A summary of ECP treatments is shown in Table 1.

Interestingly, despite significant clinical improvement, biopsies after completion of ECP treatment did not reveal significant changes compared to biopsies prior to ECP (Figures 1(c) and 1(d)). The benefit of ECP appeared to plateau 14 months after the initiation of therapy.

**Figure 2:**
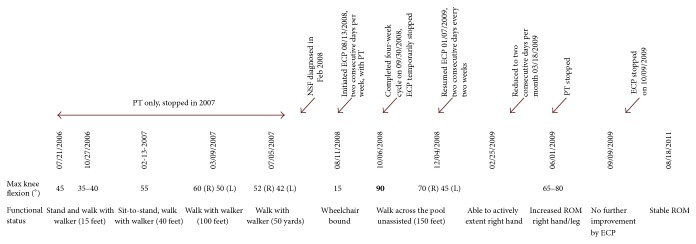
Disease and treatment timeline.

Subsequently, patient was maintained on tacrolimus alone which continued to slowly improve his symptoms. The summary of the disease course is shown in Figure 2.

## 3. Discussion

Exceptional reviews with visual aids are highly recommended for further reading on the general aspects of NSF [6, 7]. For this case specifically, we were initially impressed with the rapid onset of improvements in this patient. This was largely a result of comparing this patient to our usual experience with photopheresis in the treatment of chronic graft-versus-host disease after hematopoietic progenitor cell transplant in which improvements tend to be relatively slower and require at least a few to several months of ECP to gauge benefit.

Upon further reading, we discovered that the rapidity of improvement is not necessarily unique to this patient nor is it necessarily that rapid compared to other reports. Kafi et al. reported that a patient treated with phototherapy (a similar but not identical modality to ECP) and that “softening of the patient's skin lesions was first noted during the second week of therapy” [4].

More specific to ECP, Gilliet et al. reported 3 NSF patients treated with ECP [5]. They reported that “[a]ll three patients showed a softening of the skin lesions and a marked improvement of the joint motility starting after four cycles of ECP” and that “after two cycles of therapy, [one] patient noted a marked softening of her lesions on the lower leg.”

Thus, if a patient improves from ECP, then it would not be unexpected to see such benefits within approximately 2–6 weeks. This is speculated to depend upon many variables including the frequency of ECP, duration of ECP, other additional treatments, heterogeneity of disease, and interpatient biological heterogeneity.

## 4. Conclusion

We report a case of NSF treated with ECP over a period of 14 months which demonstrated significant movement improvement that was sustained after cessation of ECP. ECP may be useful for NSF patients with longstanding disease. It is important to keep in mind that clinical improvement may not be proportional to histological improvement, and the ideal ECP regimen is highly personalized. For further characterization of the benefit of ECP in NSF patients, standard methods to assess disease improvement are needed.

## Figures and Tables

**Table 1:** Summary of ECP treatments.

One cycle q week × 4	
(three-month interval without ECP)	
One cycle q2 weeks × 6	
One cycle q month × 7	

*Note.* One “cycle” equals ECP on two consecutive days.
